# Iridin Induces G2/M Phase Cell Cycle Arrest and Extrinsic Apoptotic Cell Death through PI3K/AKT Signaling Pathway in AGS Gastric Cancer Cells

**DOI:** 10.3390/molecules26092802

**Published:** 2021-05-10

**Authors:** Pritam-Bhagwan Bhosale, Preethi Vetrivel, Sang-Eun Ha, Hun-Hwan Kim, Jeong-Doo Heo, Chung-Kil Won, Seong-Min Kim, Gon-Sup Kim

**Affiliations:** 1 Research Institute of Life Science and College of Veterinary Medicine, Gyeongsang National University, Gazwa, Jinju 52828, Korea; shelake.pritam@gmail.com (P.-B.B.); preethivetrivel05@gmail.com (P.V.); sangdis2@naver.com (S.-E.H.); shark159753@naver.com (H.-H.K.); wonck@gnu.ac.kr (C.-K.W.); 2 Biological Resources Research Group, Bioenvironmental Science & Toxicology Division, Korea Institute of Toxicology (KIT), 17 Jeigok-gil, Jinju 52834, Korea; jdher@kitox.re.kr

**Keywords:** iridin, flavonoid, gastric cancer, extrinsic apoptosis, PI3K/AKT pathway

## Abstract

Iridin is a natural flavonoid found in *Belamcanda chinensis* documented for its broad spectrum of biological activities like antioxidant, antitumor, and antiproliferative effects. In the present study, we have investigated the antitumor potential of iridin in AGS gastric cancer cells. Iridin treatment decreases AGS cell growth and promotes G2/M phase cell cycle arrest by attenuating the expression of Cdc25C, CDK1, and Cyclin B1 proteins. Iridin-treatment also triggered apoptotic cell death in AGS cells, which was verified by cleaved Caspase-3 (Cl- Caspase-3) and poly ADP-ribose polymerase (PARP) protein expression. Further apoptotic cell death was confirmed by increased apoptotic cell death fraction shown in allophycocyanin (APC)/Annexin V and propidium iodide staining. Iridin also increased the expression of extrinsic apoptotic pathway proteins like Fas, FasL, and cleaved Caspase-8 in AGS cells. On the contrary, iridin-treated AGS cells did not show variations in proteins related to an intrinsic apoptotic pathway such as Bax and Bcl-xL. Besides, Iridin showed inhibition of PI3K/AKT signaling pathways by downregulation of (p-PI3K, p-AKT) proteins in AGS cells. In conclusion, these data suggest that iridin has anticancer potential by inhibiting PI3K/AKT pathway. It could be a basis for further drug design in gastric cancer treatment.

## 1. Introduction

Gastric cancer is the fifth most prevalent cancer that develops in the gastrointestinal tract and contributes to the second leading cause of mortality globally [1]. The primary cause of gastric cancer is *Helicobactor pylori* infection; other secondary factors include smoking and consumption of salty and fermented foods [2]. Surgical procedures are the popular treatments for advanced stages of gastric cancer. In addition, diverse options such as neoadjuvant chemotherapy, targeted therapy, immunotherapy, and radiation therapy are practiced [3]. Treatment with chemotherapeutic agents has many adverse side effects, such as nausea, vomiting, hair loss, and appetite loss [4]. So, appropriate diagnosis, prognosis, and review of the response of cancer patients to treatment are essential to ensure successful medication and mitigate the harmful effects of the treatment [5,6].

In recent times, the advent of preclinical and clinical studies has supported the belief that dietary flavonoids have potentially beneficial effects on various health conditions, including cancer [7]. The type I form of programmed cell death called apoptosis is divided into two main pathways: extrinsic and intrinsic apoptotic pathway. The extrinsic apoptosis pathway begins with the activation of the death ligand-receptor system, while the intrinsic apoptotic pathway begins within mitochondria, leading to apoptosis formation [8,9]. Eventually, both the pathways contribute to the activation of different Caspase proteins, caspase-9 in case of intrinsic apoptosis and caspase-8 in extrinsic mediated pathway. Both the pathways converge to cleave polymer adenosine diphosphate ribose (PARP), which leads to DNA damage, induced cell death mechanisms [10]. The deregulation of cell-cycle proteins can lead to uncontrolled cell growth and spread, which eventually initiates tumor growth [11]. Flavonoids exhibit suppressed proliferation rate of cancer cells through the induction of cell cycle arrest [12].

PI3K/AKT signaling pathway is one of the essential intracellular pathways that regulate survival, cell growth, differentiation, and cellular metabolism [13,14]. The components of this pathway are often irregular in a variety of tumors, making it a preferred candidate for anticancer therapy [15]. Recent studies showed inhibition of PI3K/AKT signaling pathway by flavonoid treatment that controls the apoptosis in human cancer cells in vitro [16].

Natural flavonoids have received considerable attention as substitutes for developing novel antineoplastic agents in recent years [17]. Polyphenols are a group of organic compounds involving flavonoids (e.g., flavanols, flavones, anthocyanins, isoflavonoids, flavonols, and flavanones) and nonflavonoids (e.g., xanthones, lignans, phenolic acids, tannins, and stilbenes) [18]. Flavonoids are secondary metabolites consisting of a benzopyrone ring with phenolic or poly-phenolic groups attached at different positions and are abundant in fruits, flowers, seeds, stems, cereals, nuts, vegetables, and herbs [19]. Documented studies explained that the dietary intake of flavonoids is capable of demonstrating protective effects against cancer risk [20,21]. Flavonoids have a wide range of anticancer activities that modulate ROS-scavenging enzyme activity, autophagy, and apoptosis that inhibits cancer cell proliferation, invasion, and cell cycle arrest [22]. Iridin is a glycoside flavonoid monomer present in various plant species like *Belamcanda chinensis*, *Iris kumaonesis,* and *Iris florentina* [23]. Compounds from *Belamcanda chinensis* have a reported diverse variety of biological activities such as antitumor, antioxidant, and antiproliferative effects [24,25,26].

In the current study, we investigated the anticancer effects of iridin in human gastric cancer AGS cells, and the molecular events have been explored. We report that iridin suppresses cell proliferation, induces G2/M phase cell cycle arrest, and exhibits Fas-mediated extrinsic apoptotic cell death by inhibiting PI3K/AKT signaling in AGS cells.

## 2. Results

### 2.1. Iridin Inhibits Cell Proliferation and Triggers Cell Death in AGS Cells

**Figure 1:**
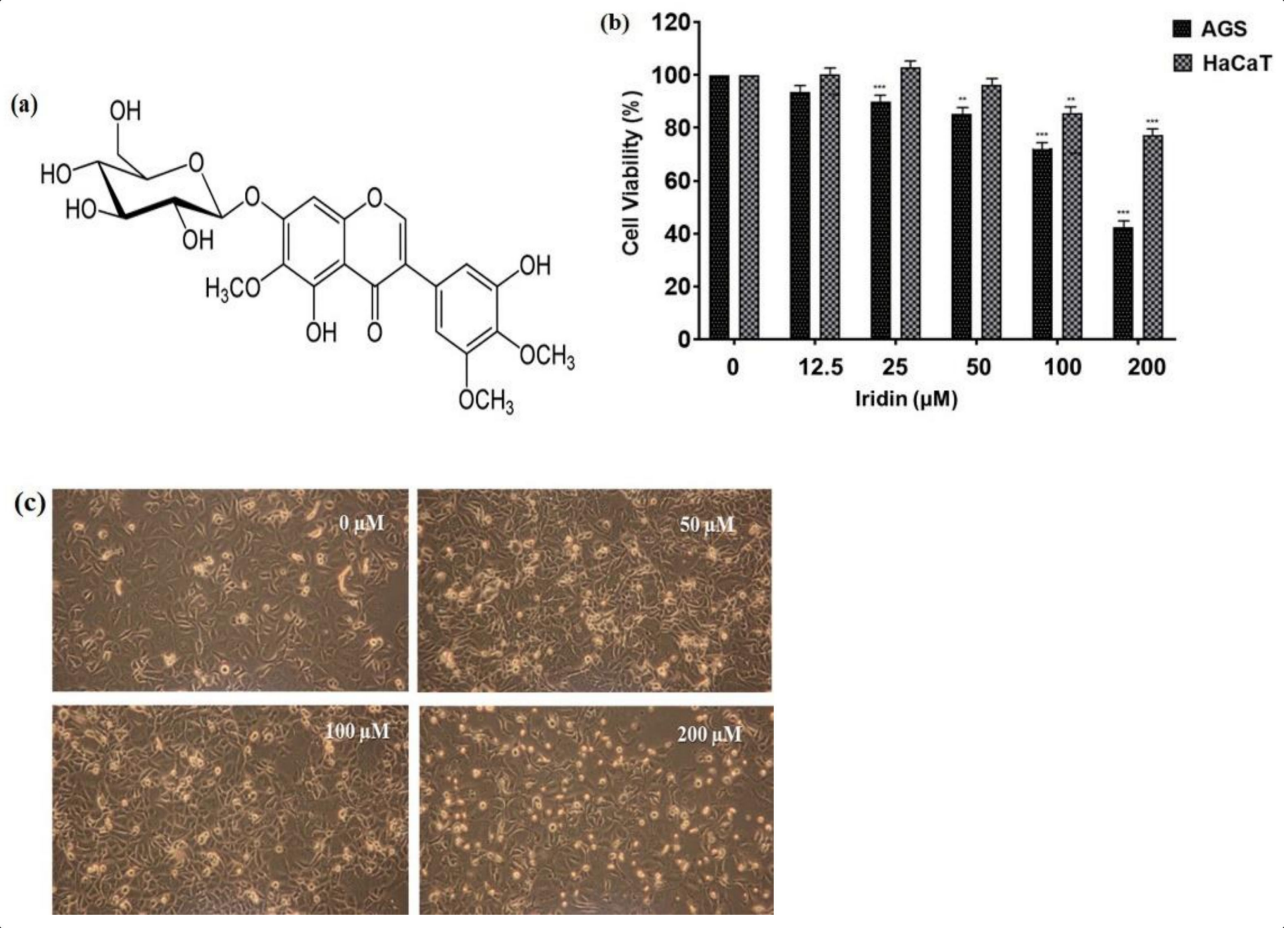
Effect of iridin on the viability of AGS cells. (**a**) Structure of iridin. (**b**) Determination of cytotoxicity of AGS and human keratinocyte HaCaT cells after treatment with iridin for 48 h at various concentrations (0, 12.5, 25, 50, 100, and 200 µM) was measured by 3-(4,5-dimethylthiazol-2-yl)-2,5-diphenyltetrazolium bromide (MTT) assay. (**c**) Morphological changes of AGS cells were visualized under light microscopy (×400) after treatment with iridin. Bar values represent the mean, and the error bar represents the standard error of the mean (SEM) of three separate experiments. ** *p* < 0.01 vs. control, *** *p* < 0.001 vs. control.

The chemical structure of iridin consists of a 7-glucoside of irigenin (Figure 1a). The cytotoxicity of iridin on AGS and HaCaT cells was determined by 3-(4,5-dimethylthiazol-2-yl)-2,5-diphenyltetrazolium bromide (MTT) assay at different iridin concentrations (0, 12.5, 25, 50, 100, and 200 µM) for 48 h. Results showed that Iridin treatment does not affect cell viability in HaCaT cells as shown in (Figure 1b). We observed the concentration-dependent inhibitory effect of iridin on AGS cell viability compared with the control (0 µM) at 48 h (Figure 1b). The half-maximal inhibitory concentration (IC50) in AGS cells was 161.3 µM. HaCaT cell viability did not affected much till 200 µM after iridin treatment which is higher concentration adopted further in the study. Microscopic examination showed morphological changes in iridin-treated cells for 48 h, including floating dead cells, cell shrinkage, and reduced cell numbers (Figure 1c). These results suggested that iridin can inhibit proliferation and induce cell death in AGS cells.

### 2.2. Iridin Induces G2/M Phase Cell Cycle Arrest by Modulating Cyclin B1, Cdc25C, and CDK1 Protein Expression in AGS Cells

**Figure 2:**
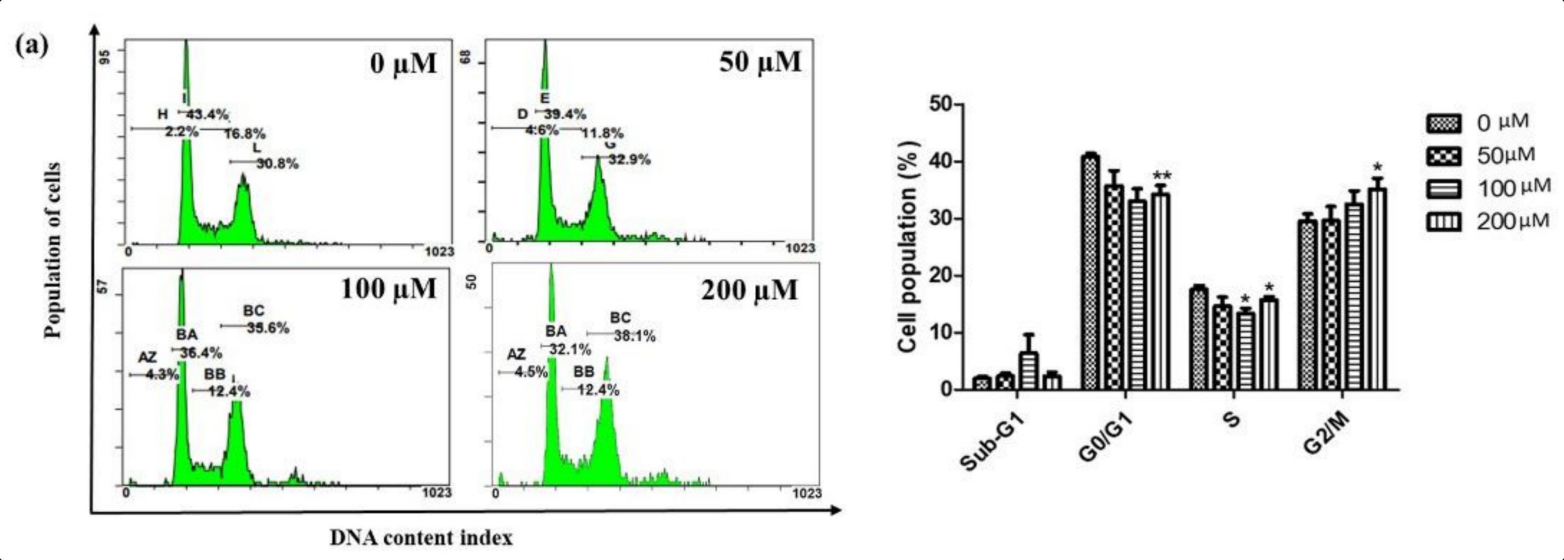

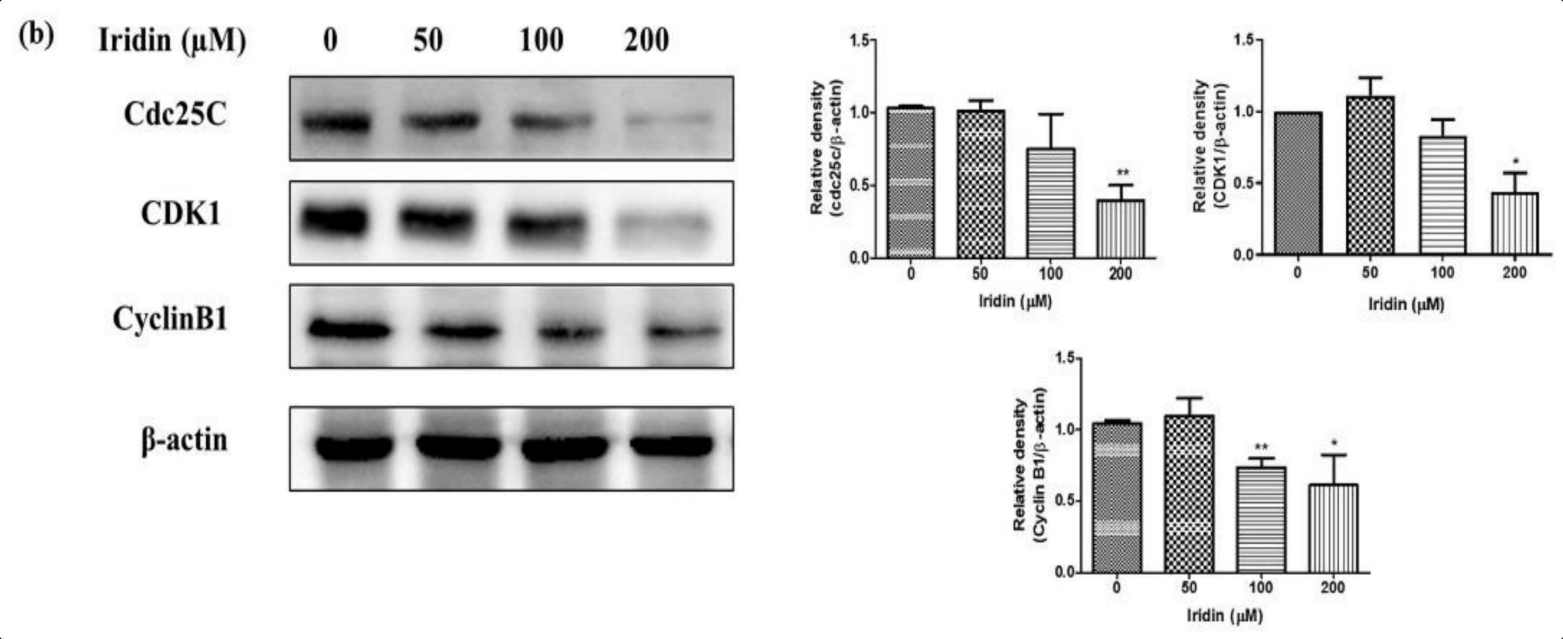
Iridin induces G2/M cell cycle arrest of AGS cells. (**a**) Flow cytometry was applied to evaluate the cell cycle distribution. The cells were treated with the indicated concentration of iridin (0, 50, 100, and 200 µM) for 48 h and treated with propidium iodide (PI) stain. (**b**) Effect of iridin on cell cycle-related proteins (Cdc25C, CDK1, Cyclin B1) were measured by immunoblot. Cells were treated with iridin (0, 50, 100, and 200 µM) for 48 h. Cell lysate were subjected to sodium dodecyl sulfate (SDS)-polyacrylamide gels and analyzed by western blot. The proteins were visualized using the detection system of Electrochemiluminescence (ECL). Bar and error bar values represent the mean ± standard error of the mean (SEM) of three separate experiments. * *p* < 0.05 vs. control, ** *p* < 0.01 vs. control.

Since iridin suppressed AGS cell growth, we investigated the cell cycle arrest in AGS cells by flow cytometry in iridin-treated cells. Precise evaluations of cellular DNA content through flow cytometry classify the phases of the cell cycle. Iridin-treated cells were stained with propidium iodide (PI), and DNA integrity was analyzed using flow cytometry. As shown in Figure 2a, there was a considerable amount of cell aggregation treated with iridin were found at the G2/M phase and only moderate changes were found in the S and G1 stage. To know the possible molecular mechanisms implicated with iridin-induced growth arrest in AGS cells, regulatory proteins of the cell cycle were examined by western blot analysis. The immunoblot result showed that the cells treated with iridin showed reduced Cyclin B1, CDK1, and Cdc25C expression, which plays a significant role in maintaining the G2/M phase (Figure 2b).

### 2.3. Iridin Induces Apoptosis in AGS Cells

**Figure 3:**
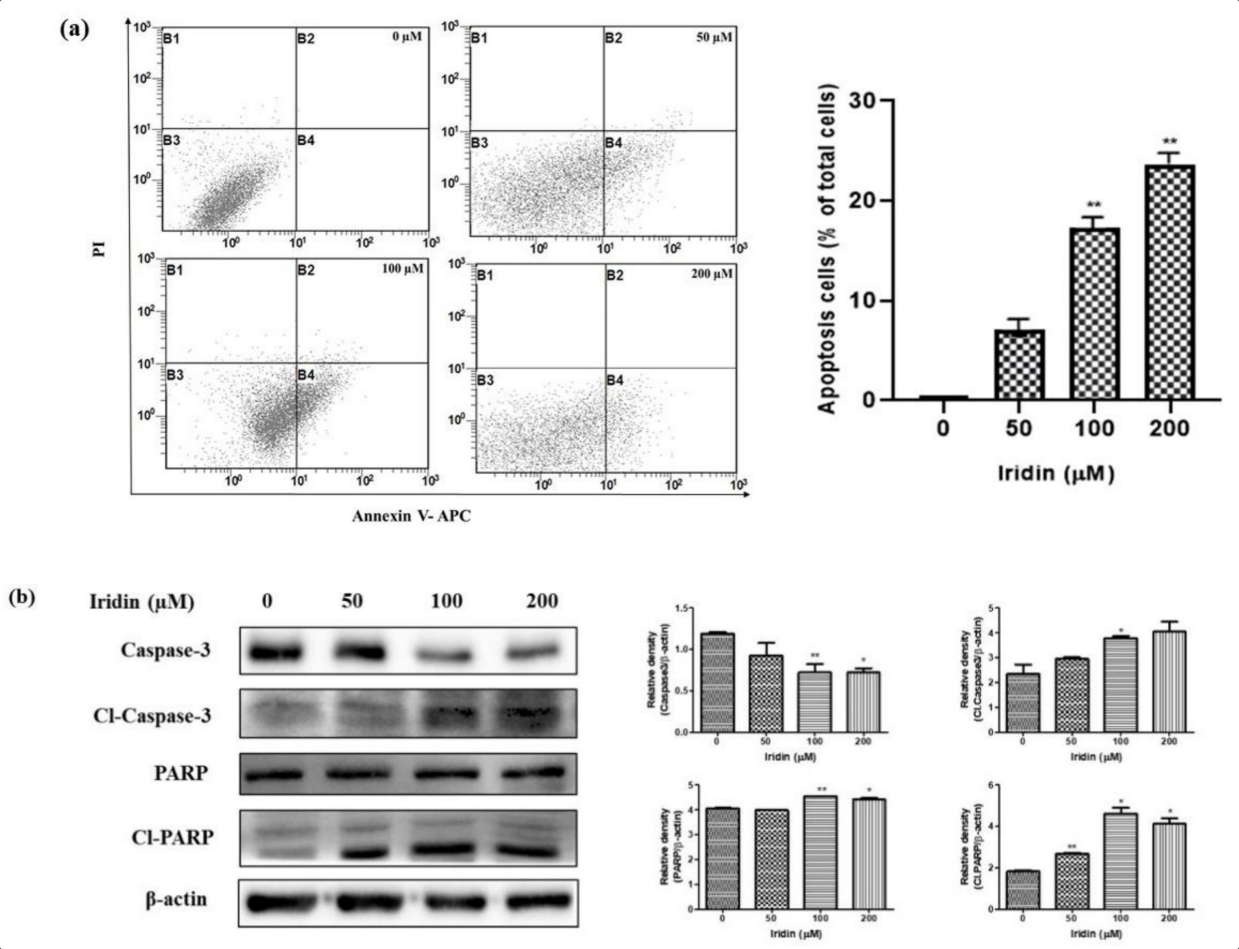
Iridin induces apoptosis in AGS cells. (**a**) AGS cells were treated with indicated concentrations with iridin (0, 50, 100, and 200 µM) for 48 h. Double staining with allophycocyanin (APC/Annexin V and propidium iodide (PI) was performed to analyze by flow cytometry. (**b**) The cells were treated with iridin at indicated concentrations (0, 50, 100, and 200 µM) for 48 h. Total cell lysates were separated in sodium dodecyl sulfate (SDS)-polyacrylamide gels and blotted onto polyvinylidene difluoride (PVDF) membranes. The membranes were probed with cleaved caspase-3 (Cl-Caspase-3), PARP, cleaved- polymeric adenosine diphosphate ribose (PARP), and β-actin antibodies. The proteins were visualized using the detection system of Electrochemiluminescence (ECL). Bar and error bar values represent the mean ± standard error of the mean (SEM) of three separate datasets. * *p* < 0.05 vs. control, ** *p* < 0.01 vs. control.

We examined the effect of iridin on apoptosis induction in AGS cells. Iridin-treated cells were double-stained with PI together with allophycocyanin (APC)/Annexin V and subjected to flow cytometry analysis. As shown in Figure 3a, iridin treatment effectively induced apoptosis at an increasing rate of 50, 100, and 200 µM, respectively. Moreover, iridin induces early apoptosis in AGS cells. These findings were collaborated with immunoblot assay showed upregulation of cleaved PARP and cleaved Caspase-3 (Cl- Caspase-3) in a concentration-dependent manner (Figure 3b), the hallmark mechanism of apoptosis. These data indicate that iridin induces apoptotic cell death in AGS cells.

### 2.4. Iridin Induces Extrinsic Apoptotic Pathway

**Figure 4:**
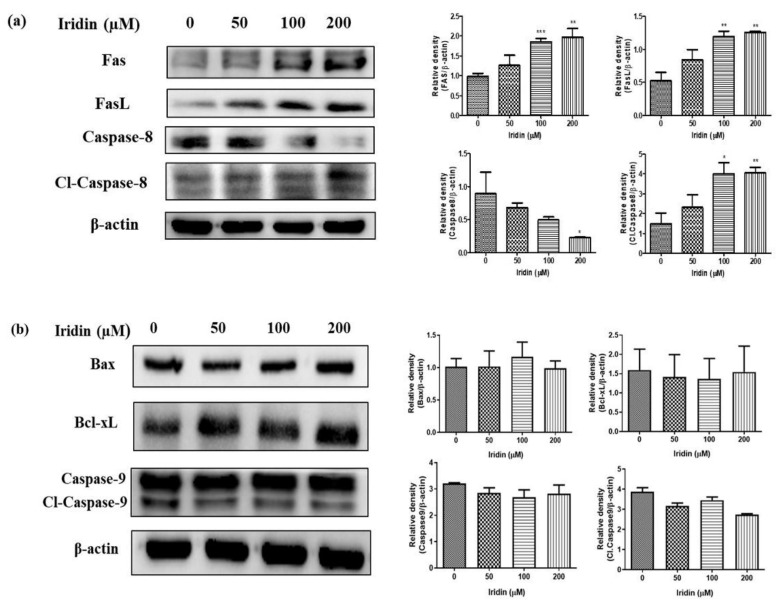
Iridin induces an extrinsic apoptotic pathway in AGS cells. (**a**) The cells were treated with iridin at indicated concentrations (0, 50, 100, and 200 µM) for 48 h. Total cell lysates were separated in sodium dodecyl sulfate (SDS)-polyacrylamide gels and blotted onto polyvinylidene difluoride (PVDF) membranes. The membranes were probed with Fas, FasL, caspase-8, cleaved caspase-8 (Cl-Caspase-8), and β-actin antibodies. (**b**) The cells were treated with iridin at indicated concentrations (0, 50, 100, and 200 µM) for 48 h. Total cell lysates were separated in sodium dodecyl sulfate (SDS)-polyacrylamide gels and blotted onto polyvinylidene difluoride (PVDF) membranes. The membranes were treated with antibodies for Bcl-xL, Bax, Caspase-9, cleaved Caspase-9 (Cl-Caspase-9), and β-actin. Electrochemiluminescence (ECL) detection system used to visualize the proteins. Bar and error bar values represent the mean ± standard error of the mean (SEM) of three independent experiments. * *p* < 0.05 vs. control, ** *p* < 0.01 vs. control, *** *p* < 0.001 vs. control.

Fas ligand (FasL) is an apoptotic ligand and activates the extrinsic apoptotic pathway. To find the mechanism of iridin-mediated apoptosis, we evaluated extrinsic and intrinsic apoptotic pathways. Expression of Fas and FasL was assessed by western blot analysis. Immunoblot results suggested that the Fas and FasL upregulated in a dose-dependent manner, followed by reducing Caspase-8 and Caspase-3 and activation of cleaved Caspase-8 (Cl-Caspase-8) (Figure 4a). Further, we analyzed the antiapoptotic protein Bcl-xL, pro-apoptotic protein Bax, and the mitochondrial apoptotic pathway-associated protein Caspase-9 in iridin-treated AGS cells by immunoblot. As shown in Figure 4b, the expression levels of Bax, Bcl-xL, and Caspase-9 in the AGS cells treated with iridin did not change. This data demonstrates that the intrinsic mediated pathway is not involved in iridin-induced apoptosis in AGS cells. Cell death in iridin-treated AGS cells occurs via the extrinsic apoptotic pathway.

### 2.5. Effect of Iridin on PI3K/AKT Signaling Pathway in AGS Cells

**Figure 5:**
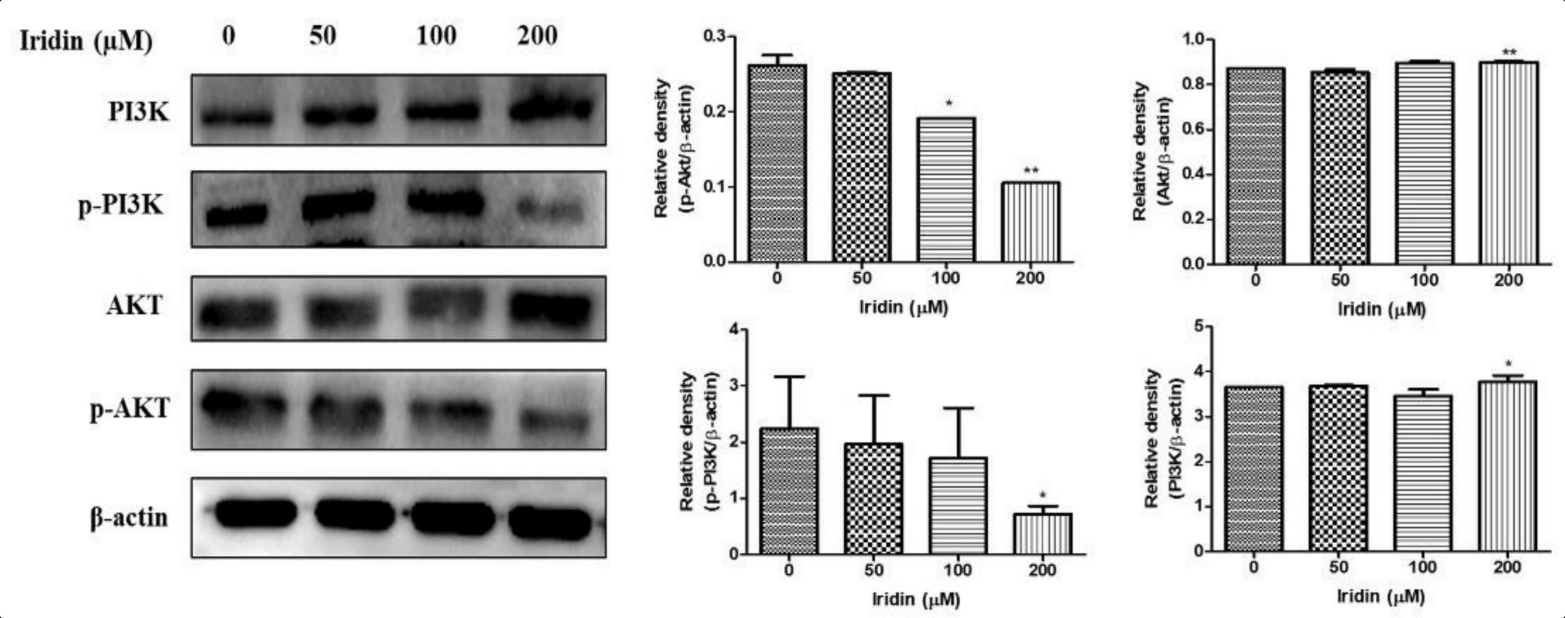
Effect of iridin on inhibition of PI3K/AKT pathway. AGS cells were treated with the indicated concentration of iridin (0, 50, 100, and 200 µM) for 48 h. The cell lysate was subjected to sodium dodecyl sulfate (SDS)-polyacrylamide gels and transferred onto polyvinylidene difluoride (PVDF) membranes. Following membranes were probed to PI3K, p-PI3K, AKT, p-AKT, and β-actin antibodies. Electrochemiluminescence (ECL) detection system used to visualize the proteins. Bar and error bar values represent the mean ± standard error of the mean (SEM) of three separate datasets. * *p* < 0.05 vs. control, ** *p* < 0.01 vs. control.

Western blot analysis was used to examine the inhibitory effect of iridin on the PI3K/AKT signaling pathway proteins. We evaluated the expression levels of proteins involved in the PI3K/AKT signaling pathway in AGS cells by immunoblot analysis. As shown in Figure 5, p-AKT and p-PI3K expression were reduced in a dose-dependent manner at 48 h with no change in the total expression of these proteins. Together, these findings demonstrated that iridin regulates apoptosis in AGS cells via the PI3K/AKT pathway.

## 3. Discussion

Clinical studies showed that dietary flavonoid consumption might reduce the risk of colon, pancreatic, lung, and prostate tumors. The generalization of this anticancer action of flavonoids is a subject of study, as some of the advantages are unique to subclasses of flavonoids [20]. *Belamcanda chinensis* is a member of the *Iridaceae* family, which is widely distributed in eastern Asia. It has been used in traditional Chinese medicine as anti-inflammatory, expectorant, anticancer, and throat disorders [25,27,28]. Phytoestrogens derived from *Belamcanda chinensis* like tectorigenin and irigenin have an antiproliferative and antitumor effect on various cancer cells [24]. An extract from Korean *Scutellaria baicalensis* Georgi, which contains many flavonoids, including iridin, induces apoptosis in AGS and in fibrosarcoma cells [29,30]. Though, the effect of iridin on gastric cancer cells remains elusive. Herein, this study provides an overview to understand the impact of iridin in gastric cancer cells.

In the current study, investigations were performed on the anticancer effect and its mechanism of action using human gastric cancer AGS cells. The cytotoxic assessment of iridin was primarily determined in AGS cells at different concentrations, and three fixed dosages were adopted throughout the study. The cytotoxic evaluation showed that iridin suppressed cell proliferation in AGS cells in a concentration-dependent manner at 48 h treatment. Typical cell death-related morphological features such as cell blebbing, rounding, and cell shrinkage were observed on iridin-treated cells.

Due to the development of effective treatments for cancer characterized by abnormalities in cell death regulation, it is necessary to understand the different cell death mechanisms [31]. The evasion of apoptosis in malignant cells is a hallmark of cancer. The induction of apoptotic cell death by anticancer agents is a promising strategy adopted for developing chemotherapeutics to treat various cancers [32]. Apoptosis is one type of programmed cell death that leads in an orderly fashion of removing damaged cells [33]. In the present study, upon confirmation of cytotoxicity by iridin, the induction of apoptosis was studied through double staining (APC/Annexin V and PI) showed increased cell death fraction at the early stage of apoptosis.

Our investigation of cell cycle distribution revealed that G2/M phase cell cycle arrest was observed in iridin-treated AGS cells which were supported by downregulation of cell cycle proteins Cyclin B1, CDK1, and Cdc25C through western blot, which play a crucial role in maintaining the G2/M phase. Cyclin B-CDK1 activity is specific to the G2/M phase checkpoint. Accumulation of cyclin B increases the activity of cyclin-dependent kinase CDK1, and cyclin B-Cdc2 activates Cdc25C. Inhibition of CyclinB1, CDK1, and Cdc25C cell cycle proteins causes arrest in the G2/M phase. Previous studies also showed that flavonoids such as scutellarein and apigetrin induced G2/M phase cell cycle arrest and apoptosis in Hep3B and AGS cells, respectively [13,34].

To elucidate the molecular mechanism mediated by iridin for apoptosis induction, immunoblotting was performed on the apoptotic pathway marker proteins. To develop effective therapies for cancer treatment, it is necessary to study the abnormalities in cell death regulation and understand different pathways in which cells lose viability and ultimately die [35]. The extrinsic apoptotic pathway begins when the death-ligand binds to a death receptor which forms a death-inducing signaling complex (DISC) [36]. FasL binding promotes Fas clustering and its binding to FADD in the Fas-mediated apoptosis pathway. FADD recruits Caspase-8 and Caspase-10 to form the DISC [37]. Bax/Bcl-xL is considered an important parameter in cell death through the intrinsic apoptotic pathway. The intrinsic pathway involves various stimuli that act on multiple targets within the cell, which causes activation of cytochrome C and pro-apoptotic proteins to induce cell death [35]. Iridin treatment significantly elevated the levels of extrinsic apoptotic pathway proteins such as FasL and Fas, followed by Caspase-8 and Caspase-3, including accumulation of PARP expression.

In contrast, iridin does not affect mitochondrial apoptotic pathway-related proteins like Bcl-xl, Bax, and Caspase-9. Thus, our result shows iridin-mediated cell death in AGS cells is triggered by the activation of extrinsic apoptotic pathway with no intrinsic pathway involvement. Some previous studies also confirm that flavonoids like scutellarein and poncirin also cause extrinsic apoptotic cell death in cancer cells [34,38]. Similarly, dihydrochalcone derivative and hesperetin induce extrinsic apoptosis in breast cancer cells and lung cancer cells, respectively [39,40].

PI3K/AKT signaling cascades is an essential and well-studied pathway in cells due to their associations with normal physiologic and pathophysiological processes playing a diverse array of functions [41]. When activated abnormally, it can activate downstream signaling molecules, thus further affecting the occurrence and development of various malignant tumors [42]. We examined the expression of proteins through western blot analysis, which shows iridin considerably reduced p-AKT and p-PI3K expression in AGS cells. Similar to the present result, previous evidence suggests that flavonoids induced inhibition of the PI3K/AKT pathway in different cancer cell lines [43,44,45].

## 4. Materials and Methods

### 4.1. Chemicals and Reagents

RPMI-1640 medium, DMEM and fetal bovine serum (FBS) obtain from Gibco (BRL Life Technologies, Grand Island, NY, USA). Propidium iodide (PI) and 3-(4,5-Dimethylthiazol-2-yl)-2,5-diphenyltetrazolium bromide (MTT) purchased from Sigma-Aldrich (St. Louis, MO, USA). Primary antibodies obtained from Cell Signaling Technology (Danvers, MA, USA) for various proteins including Cyclin B1 (Cat. No. 4138S), Cdc25c (Cat. No. 4688S), Bcl-xL (Cat. No. 2764S), Bax (Cat. No. 5023S), caspase 3 (Cat. No. 9662S), Cl-Caspase-3 (Cat. No. 9664S), Caspase-9 (Cat. No. 9502S), Poly ADP-ribose polymerase (PARP; Cat. No. 9542S), Cleaved-PARP (Cat. No. 5625S), FasL (cat.no. 68405S), Fas (Cat. No. 4233S), caspase-8 (Cat. No. 4190S), Cl-Caspase-8 (Cat. No. 9496S), p-PI3K (Cat. No. 4228S), PI3K (Cat. No. 3011S), p-AKT (Cat. No. 4060S), AKT (Cat. No. 2920S), and β-actin (Cat. No. 4970S). The primary antibodies for CDK1 (Cat. No. ab131450) were purchased from Abcam (MA, USA). Horseradish peroxidase (HRP)-conjugated secondary antibodies were obtained from Bethyl Laboratories, Inc (Montgomery, TX, USA, anti-rabbit, A120-101P; and antimouse, A90-116P).

### 4.2. Cell Culture and Iridin Treatment

Human gastric cancer cell line AGS and human keratinocyte HaCaT cells were acquired from the Korea Cell Line Bank (Seoul, Korea). The AGS cells were cultured in RPMI-1640 medium supplemented with 10% (*v*/*v*) heat-inactivated FBS and 1% penicillin/streptomycin in a humidified atmosphere with 5% CO_2_ at 37 °C. We culture AGS cells for not more than 15 passages. Iridin was purchased from (ChemFaces, Wuhan, China). Cells were grown at a confluence of 80% either untreated (DMSO) or treated in complete media with specified iridin concentrations (0, 50 100, and 200 µM) for 48 h.

### 4.3. Cell Viability Assay

The AGS (5 × 10^4^), cells and HaCaT (1 × 10^4^) cells were seeded in a 48 well plate and treated with different concentrations of iridin (0, 12.5, 25, 50, 100, and 200 µM). Further, treated cells were incubated for 48 h at 37 °C. Cell viability was assessed using MTT assay. After incubation, 50 µL of 0.5% (*w*/*v*) MTT dissolved in 1x PBS was added to each well and incubated for about three hours at 37 °C. Formazan precipitates formed after incubation was dissolved in 300 µL of DMSO and calculated the absorbance of transformed dye at 540 nm with a microplate reader (BioTek, Winooski, VT, USA). Cell viability was expressed as a percentage of proliferation versus iridin untreated group.

### 4.4. Analysis of Cell Cycle Distribution Using Flow Cytometry

Flow cytometry technique used to analyze the progression of the cell cycle and cell death. The AGS cells were incubated with or without iridin at 50, 100, and 200 µM concentrations for 48 h at 37 °C. The cells were washed with ice-cold PBS after incubation, trypsinized, collected in a 15 mL conical tube, and pelleted by centrifugation (3000 rpm) for 4 min. The pellets were washed twice with ice-cold PBS and fixed in 70% ice-cold ethanol for one hour at −20 °C. Further, the cell suspension was centrifuged, and the cells were washed in PBS and re-suspended in 400 µL of PBS containing 50 µg/mL PI (Sigma-Aldrich, St. Louis, MO, USA) and 0.1mg/mL RNase A and incubated in the dark for 15 min at room temperature. Flow cytometry analyses were performed with Cytomics FC 500 (Beckman Coulter, Brea, CA, USA). In each sample, approximately 10,000 cells were sorted. The data obtained were analyzed by using CXP Software (Beckman Coulter, Inc., Fullerton, CA, USA)

### 4.5. Annexin V Propidium Iodide Apoptosis Detection

The cell death population of iridin-treated AGS cells was calculated by allophycocyanin (APC)/annexin V apoptosis detection kit according to the manufacturer’s protocol (BD Biosciences, San Diego, CA, USA). Cells were seeded on 60-mm plates at 1 × 10^5^ cells and incubated with specified concentrations of iridin (0, 50, 100, and 200 µM) for 48 h. The cells were harvested using trypisinization for 3 min further washed with PBS and resuspended in binding buffer that comes along with the kit. The cells were stained at room temperature with APC/annexin V and PI for 15 min in the darkroom before binding buffer addition. Flow cytometry analyses were performed with Cytomics FC 500 (Beckman Coulter, Brea, CA, USA). In each sample, approximately 10,000 cells were sorted. The data obtained were analyzed using CXP software (Beckman Coulter, Inc., Fullerton, CA, USA).

### 4.6. Western Blotting Analysis

The protein expression of AGS cells after treatment with iridin was implemented by western blot analysis. Cells were seeded at 3 × 10^5^ in 60-mm per plate treated with specified concentrations of iridin (0, 50, 100, and 200 µM) for 48 h. Then the harvested cells were lysed in radioimmuno-precipitation assay (RIPA) buffer (iNtRON Biotechnology, Seoul, South Korea) consisting of protease inhibitor and phosphatase inhibitor cocktail (Thermo Fisher Scientific, Rockford, IL, USA) for 30 min in ice. For protein quantification, BCA (Thermo Fisher Scientific, Rockford, IL, USA) assay method was used. The 8–15% SDS-polyacrylamide gel electrophoresis (SDS-PAGE) was used to separate the proteins and transferred on the membrane of polyvinylidene difluoride PVDF (Immunobilon-P, 0.45 mm; Millipore, Billerica, MA, USA) using a semi-dry transfer method (Atto Corp., Tokyo, Japan). The membranes were blocked using bovine serum albumin (BSA) with tris-buffered saline, including 1% tween 20 (TBS-T, pH 7.4) at 4 °C overnight 1:1000 dilution of respective primary antibody concentration. The membranes were washed five times with TBS-T for 10 min intervals and incubated for 3 h at RT with a dilution (1:1000 to 1:5000) of the HRP-conjugated secondary antibody. Further, the membranes were washed with TBS-T five times. The blots were developed under an electrochemiluminescence (ECL) detection system (Bio-Rad Laboratory, Hercules, CA, USA). The quantification of proteins was analyzed using the ImageJ software program (U.S. National Institutes of Health, Bethesda, MD, USA). The protein band densitometry readings were standardized by comparison with the β-actin expression.

### 4.7. Statistical Analysis

All the data were expressed as the mean ± standard error of the mean (SEM) of three independent experiments. Significant differences between groups were estimated by one-way factorial analysis of variance (ANOVA) followed by Bonferroni test. The *p* value < 0.05 was considered statistically significant.

## 5. Conclusions

**Scheme 1:**
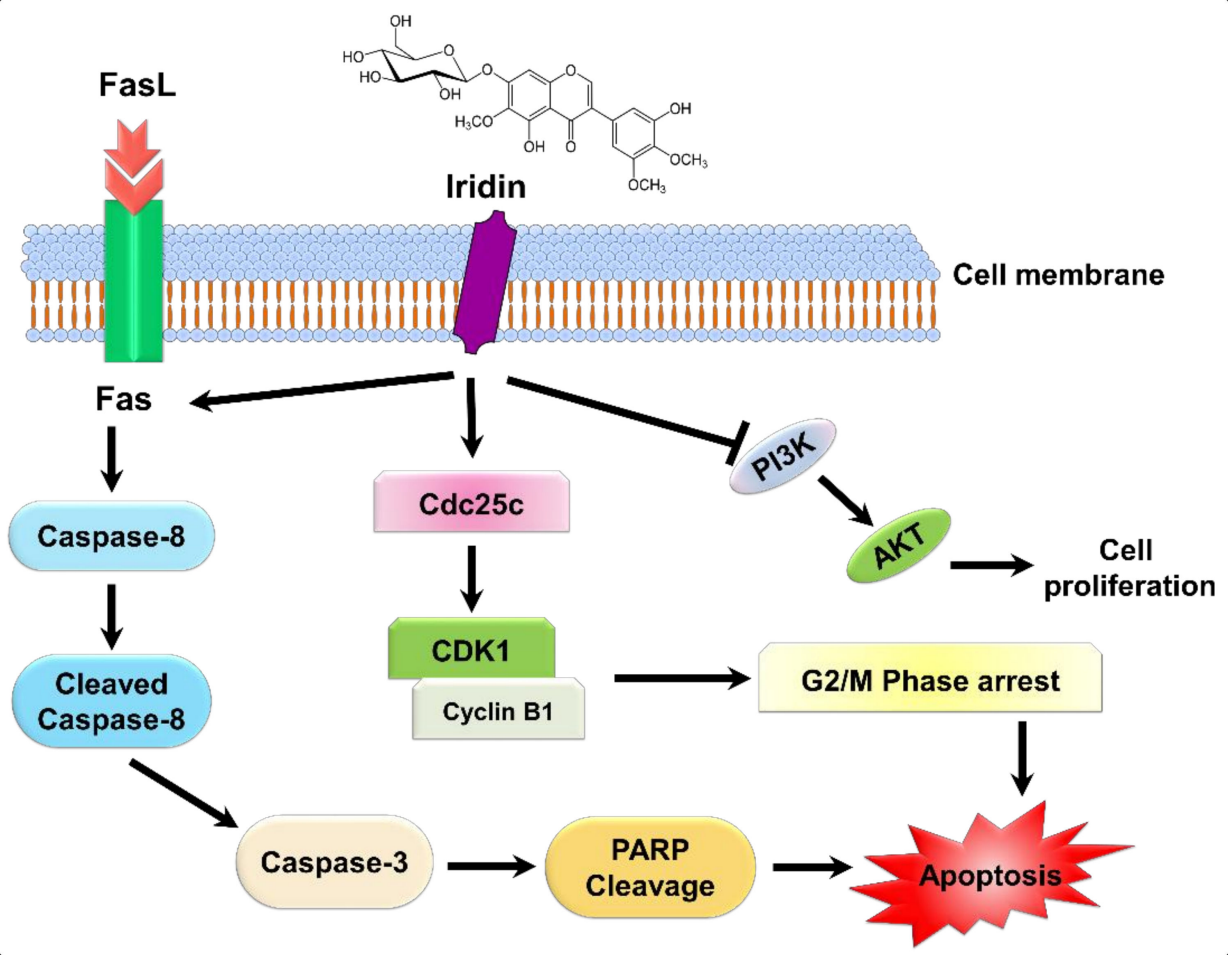
Schematic diagram representing the anticancer mechanism of iridin on AGS cells through induction of G2/M phase cell cycle arrest, apoptosis by inhibition of PI3K/AKT pathway.

In summary, the study indicates the anticancer effect of iridin and its mechanism of action in inducing apoptotic cell death in AGS gastric cancer cells. Iridin suppressed cell growth, caused G2/M phase cell cycle arrest, and induced extrinsic mediated apoptosis in AGS cells in vitro as shown in Scheme 1. The findings explore the significant inhibitory action of iridin on AGS cell lines and provide a basis for further development as an anticancer agent against gastric cancer.

## Data Availability

Not applicable.
